# PSMD14 Transcriptionally Activated by MEF2A Promotes Pancreatic Cancer Development by Upregulating SPON2 Expression

**DOI:** 10.1002/kjm2.70007

**Published:** 2025-03-11

**Authors:** Yun‐He Hao, Cheng‐Ru Yang, Wu‐Jiang Shi, Xiang‐Yu Zhong

**Affiliations:** ^1^ Department of Hepatopancreatobiliary Surgery The Second Affiliated Hospital of Harbin Medical University Harbin China

**Keywords:** m6A, MEF2A, pancreatic cancer, PSMD14, SPON2

## Abstract

Proteasome 26S subunit non‐ATPase 14 (PSMD14) plays a pro‐carcinogenic role in various cancers. However, its specific effects and mechanisms in pancreatic cancer (PC) remain unclear. We aimed to assess the function and mechanism of PSMD14 in PC. Fifteen paired pancreatic ductal adenocarcinoma (PDAC) tissues and adjacent non‐tumorous tissues were clinically obtained. Cell proliferation, migration, and invasion were assessed using colony formation, scratch, and Transwell assays. The interaction between the MEF2A transcription factor and the PSMD14 promoter verified by chromatin immunoprecipitation (ChIP) or dual luciferase assay. The interaction between RBM15B and SPON2 mRNA was validated by RNA immunoprecipitation (RIP) assay. The interaction between the proteins PSMD14 and RBM15B was detected by co‐immunoprecipitation (Co‐IP) assay. The m6A level of SPON2 was detected by methylated RNA immunoprecipitation (MeRIP, a common method for detecting m6A levels of mRNAs). The ubiquitination level of RNA‐binding motif protein 15B (RBM15B) was detected using Co‐IP. The role of PSMD14 in PC was further explored subcutaneous and lung metastasis models. PSMD14 was upregulated in PDAC tissues. PSMD14 knockdown inhibited PC cell viability, proliferation, migration, and invasion. MEF2A transcriptionally activated PSMD14 expression. PSMD14 knockdown promoted the ubiquitination degradation of RBM15B. Additionally, PSMD14 enhanced SPON2 mRNA stability through RBM15B‐mediated m6A modification. SPON2 overexpression impaired the effect of knockdown PSMD14. Finally, PSMD14 knockdown in PC arrested tumor growth and lung metastasis. PSMD14, transcriptionally activated by MEF2A, promotes the de‐ubiquitination of RBM15B, which upregulates SPON2 expression in an m6A‐RBM15B‐dependent manner, thereby facilitating PC proliferation, migration, and invasion.

**Abbreviations**
BCA: bicinchoninic acid
CA19‐9: carbohydrate antigen 19–9
CCK‐8: Cell Counting Kit‐8
ChIP: chromatin immunoprecipitation
Co‐IP: co‐Immunoprecipitation
CTNNB1: catenin beta 1
DAB: diaminobenzidine
FBS: fetal bovine serum
FOXA1: forkhead box A1
GPX4: glutathione peroxidase 4
H&E: hematoxylin and eosin
IHC: immunohistochemistry
m6A: N6‐methyladenosine
MEF2A: myocyte enhancer factor 2A
MeRIP: methylated RNA immunoprecipitation
MTORC1: mechanistic target of rapamycin kinase complex 1
MUT: mutant
PAAD: pancreatic adenocarcinoma
PC: pancreatic cancer
PDAC: pancreatic ductal adenocarcinoma
PSMD14: proteasome 26S subunit non‐ATPase 14
RBM15B: RNA‐binding motif protein 15B
RIP: RNA immunoprecipitation
RT‐qPCR: real time‐quantitative PCR
SPON2: Spondin‐2
STING: stimulator of interferon
WT: wild‐type
ZEB2: zinc finger E‐Box binding homeobox 2

## 1 Introduction

Pancreatic cancer (PC) is an exceptionally aggressive disease associated with an extremely poor prognosis [1]. PC includes subtypes such as pancreatic ductal adenocarcinoma (PDAC, 90%), acinar carcinoma, pancreaticoblastoma, and neuroendocrine tumors [2]. Globally, PC has become a critical health concern, with five‐year survival rates of approximately 10%, posing a severe threat to human life and health [3, 4]. Currently, surgery is the best treatment for PC, but even with successful surgery, local recurrence or metastasis occurs in more than 80% of patients [1]. Therefore, the search for prognostic biomarkers and therapeutic targets for PC is urgent.

Proteasome 26S subunit non‐ATPase 14 (PSMD14/RPN11/POH1) is a protein‐coding gene that has emerged as a potential prognostic marker for several cancers [5, 6]. Studies demonstrated that PSMD14 promoted the development of a variety of cancers, including neck squamous cell carcinoma, breast cancer, and myeloma [7, 8, 9]. And, it was published that PSMD14 was expressed highly in PDAC samples, and its targeting with specific small molecule inhibitors significantly reduced pancreatic tumor formation [10]. Notably, Gene Expression Profiling Interactive Analysis (GEPIA) prediction also indicated PSMD14 was upregulated in PC. However, the specific mechanism of action and potential clinical value of PSMD14 in PC remains unknown. Furthermore, mechanistic studies suggested that PSMD14 played a role in promoting cancer development by activating with transcription factors [8]. For example, the transcription factor RELA activated PSMD14, resulting in a positive PSMD14/NSD2‐RELA feedback loop that promoted the occurrence of myeloma [8]. Interestingly, this pre‐study revealed that myocyte enhancer factor 2A (MEF2A) acted as an upstream transcription factor of PSMD14, and silencing MEF2A significantly downregulated PSMD14 expression in PC cells. However, the relationship between MEF2A and PSMD14 has not been reported in the literature. The MEF2A protein belongs to the MADS‐box superfamily of eukaryotic transcription factors [11]. Research have demonstrated a substantial link between abnormal MEF2A expression and the progression of various tumor types, including colorectal cancer, hepatocellular carcinoma, and large B‐cell lymphoma [12, 13, 14]. Critically, a recent study showed that MEF2A regulated the transcriptional activities of folliculin interacting protein 1 (FNIP1) and FNIP2 to influence PC development [15]. Therefore, we hypothesized that MEF2A may influence PC progression by regulating PSMD14 transcription.

As the most abundant transcriptional modification in eukaryotes, N6‐methyladenosine (m6A) plays an important regulatory role in various aspects of RNA function, and it is critical in tumor promotion and progression [16]. In recent years, research has unveiled that numerous m6A regulators exhibit abnormal expression patterns in PC, and these aberrant expressions are closely associated with PC development [17]. Therefore, m6A‐related regulators may become new therapeutic targets and prognostic indicators for PC. RNA‐binding motif protein 15B (RBM15B, Catalyze m6A methylation modification of RNAs) belongs to the m6A recognition proteins, which can affect RNA stability, splicing, and translation by recognizing m6A modification sites [18]. Studies have confirmed that RBM15B regulates mRNA expression in an m6A modification‐dependent manner, thereby affecting tumorigenesis and progression, including liver cancer, melanoma, and lung cancer [19, 20, 21]. Currently, only one paper reported that RBM15B was susceptible to mutations in PC, but its role in PC had not been clarified [22]. Interestingly, the IntAct predictions revealed that PSMD14 and RBM15B interacted. PSMD14 is a deubiquitinating enzyme that may promote tumor progression by deubiquitinating different protein substrates [23]. For example, PSMD14 remarkably promoted the proliferation, migration, and invasion of gastric cancer cells via promoting polypyrimidine tract‐binding protein 1 (PTBP1) deubiquitination [24]. However, whether PSMD14 mediated the deubiquitination of RBM15B would need to be further explored. Crucially, Starbase predictions indicated that RBM15B was an RNA‐binding protein that interacts with SPON2 mRNA, and the Sequence‐based RNA adenosine methylation site predictor (SRAMP) also showed that SPON2 mRNA existed multiple m6A modification sites. Spondin‐2 (SPON2, also known as Mindin and DIL‐1) belongs to the Spondin family of cell matrix proteins, which recognize pathogens through the integrin family and participate in innate and adaptive immune processes [25]. Several research studies indicated that SPON2 might be used as a molecular marker for the diagnosis of various malignant tumors, such as ovarian cancer and colorectal cancer [26, 27]. However, SPON2 has not been reported on PC. Most importantly, we found that SPON2 expression was upregulated in PC through GEPIA predictions. Therefore, PSMD14 may deubiquitinate RBM15B and increase the stability of RBM15B, which upregulates SPON2 expression through m6A modification, thereby promoting PC progression.

Based on the above analysis, we proposed a hypothesis that PSMD14 is activated by MEF2A transcription, which upregulates SPON2 expression in an m6A‐RBM15B‐dependent manner, ultimately promoting PC progression. To this end, we conducted numerous trials to check the assumption and attempted to uncover the mechanisms by which they play a role in PC. These discoveries would contribute valuable insights for early PC diagnosis and potential treatments.

## 2 Materials and Methods

### 2.1 Clinical Tissues

This project was approved by the ethics committee of The Second Affiliated Hospital of Harbin Medical University (No. YJSKY2024‐427). Informed consent was written by all participants. PDAC tissues and adjacent non‐tumor tissues from 15 patients who underwent surgery were collected from the Second Hospital of Harbin Medical University. All patients had no history of other tumors, who had never received any radiotherapy or chemotherapy, and were included as PC patients by pathology and genetic confirmation. In addition, these patients also had no history of other immune diseases, and there was no serious damage to the heart, liver, kidneys, or other vital organs. Fresh tissues were fixed with paraformaldehyde (4%) or frozen in liquid nitrogen for subsequent experimental detection. According to the median PSMD14 expression was categorized into high and low expression, and the relationship between high and low PSMD14 expression and clinical characteristics [gender, age, tumor node metastasis (TNM) stage, tumor size, lymph node metastasis, distant metastasis, vascular invasion, perineural invasion, differentiation] of PDAC patients was analyzed in Table 1.

**TABLE 1:** Association between PSMD14 expression and clinicopathological characteristics of pancreatic ductal adenocarcinoma (PDAC) patients.

Clinicopathological characteristics	PSMD14			*p*
	*N* = 15	High (*n* = 7)	Low (*n* = 8)	
Gender				
Male	8	3	5	0.6193
Female	7	4	3	
Age (years)				
< 60	8	2	6	0.1319
≥ 60	7	5	2	
TNM stage				
I–II	9	2	7	0.0406*
III‐IV	6	5	1	
Tumor size (cm)				
< 4	9	3	6	0.3147
≥ 4	6	4	2	
Lymph node metastasis				
Negative	8	1	7	0.0101*
Positive	7	6	1	
Distant metastasis				
Negative	7	4	3	0.6193
Positive	8	3	5	
Vascular invasion				
Negative	5	3	2	0.6084
Positive	10	4	6	
Perineural invasion				
Negative	6	2	4	0.6084
Positive	9	5	4	
Differentiation				
Well	3	1	2	0.7363
Moderate	7	3	4	
Poor	5	3	2	

Abbreviation: TNM, tumor node metastasis.

* *p* < 0.05.

### 2.2 Cell Culture and Transfection

Cells were cultured in RPMI 1640 medium supplemented with 10% fetal bovine serum (FBS) and 1% penicillin/streptomyc with 5% CO_2_ at 37°C. Human normal pancreatic ductal epithelial cell lines (hTERT‐HPNE) and PC cell lines (Capan‐1, PANC‐1, SNU‐213, CFPAC‐1, SW1990, Aspc‐1) were obtained from the Chinese Academy of Science (Shanghai, China).

Negative control short hairpin RNA (sh‐NC) sh‐PSMD14 (CATGGACTAAACAGACATTAT), sh‐MEF2A (CCAGACCCTGATACTTCATAT), sh‐RBM15B (GAAGAAGAACACATGGTGATA), overexpression negative controls (OE‐NC), OE‐PSMD14, and OE‐SPON2 were purchased at GenePharma (Suzhou, China). The pCMV‐HA‐Ub obtained from HonorGene (Changsha, China). They were transfected into cells using Lipofectamine 3000 reagent (Thermo Fisher Scientific, Waltham, MA, USA). Then, cells were incubated at 37°C for 1 day. When transfection efficiency reached more than 80%, the cells were harvested for subsequent analysis.

### 2.3 Cell Viability Assay

Cell viability was determined by using the Cell Counting Kit‐8 (CCK‐8, Beyotime, Shanghai, China). Cells (1 × 10^4^ cells/well) were treated with 10 μL of CCK‐8 solution (10 μL/well) at 37°C for 1 h. Absorbance was then measured at 450 nm using the microplate reader (BioTek Instruments Inc. Winooski, VT, USA).

### 2.4 Cloning Formation Assay

Cells (400 cells/well) were cultured in 6‐well plates for 10–14 days at 37°C. When the colonies reached a certain size, they were fixed with 4% paraformaldehyde for 15 min. Subsequently, they were stained with 0.1% crystal violet for 30 min. The number of cell colonies per well was determined.

### 2.5 Scratch Wound Healing Assay

When the fusion rate of cells (3 × 10^5^ mL^−1^) reached 80%~90% in the 6‐well plate, a pipette tip of 200 μL was used to scratch on the cells. Serum‐free medium was then pipetted into each well. Photographs were captured with a microscope at 0 h and 24 h after scratching. Scratch areas were analyzed using Image J software (NIH, Bethesda, MA, USA).

### 2.6 Transwell Invasion Assay

Cells were trypsinized and diluted with serum‐free medium. Next, 100 μL of matrix gel was spread in the Transwell upper chamber and placed at 4°C for 30 min. Then 5 × 10^4^ cells were placed in the Transwell upper chamber, and then serum medium containing 20% FBS was supplemented in the lower chamber and incubated at 37°C for 24 h. Cells were fixed with 4% paraformaldehyde for 15 min in the lower chamber, then stained with 0.5% crystal violet dye for 30 min and the cells were observed under a microscope and counted.

### 2.7 Tumor Xenograft and Lung Metastasis

All animal experiments were approved by the Ethics Committee of The Second Affiliated Hospital of Harbin Medical University (No. YJSDW2024‐225).

Twenty‐four BALB/c‐nu/nu mice aged 5–6 weeks were acquired from Hunan Slake Jinda Laboratory Animal Co. Twelve mice were randomly assigned to the sh‐NC group and the sh‐PSMD14 group (*N* = 6). After 3 days of adaptive feeding of BALB/c‐nu/nu mice, each mouse was injected with 2 × 10^6^ PANC‐1 cells (transfected with sh‐PSMD14) in the groin area. Tumors were measured every 7 days from the date of inoculation by calipers. Mice were euthanized 28 days after tumor formation. Subcutaneous tumors were peeled off, and their weight and volume were recorded.

PANC‐1 cells (5 × 10^6^ cells) transfected with sh‐PSMD14 or sh‐NC were infused into the tail vein of BALB/c‐nu/nu mice to generate lung metastases. After 30 days, the mice were euthanized and lung tissue was removed. Part of it was fixed for immunohistochemistry (IHC) detection and H&E staining, while the other part of it was directly stored with liquid nitrogen for further experiments.

### 2.8 Bioinformatics Analysis

The GEPIA database (http://gepia.cancer‐pku.cn/about.html) predicted the differential expression of PSMD14 and SPON2 in PAAD patients, which also predicted the relationship between high and low PSMD14 expression and survival analysis with PAAD patients. Multiple high‐confidence binding sites between the transcription factor MEF2A and the PSMD14 promoter were predicted by the JASPAR (https://jaspar.elixir.no/) and hTFtarget (http://bioinfo.life.hust.edu.cn/hTFtarget#!/) databases. Interaction between PSMD14 and RBM15B proteins was predicted using the molecular interaction database (IntAct) (https://www.ebi.ac.uk/intact/home). The Starbase database (https://rnasysu.com/encori/index.php) predicted a regulatory relationship between the RNA‐binding protein RBM15B and SPON2 mRNA. The m6A modification sites in SPON2 mRNA were predicted by the SRAMP (http://www.cuilab.cn/m6asiteapp/old).

### 2.9 Chromatin Immunoprecipitation (ChIP) Assay

According to the manufacturer's instructions for the Simple ChIP Enzymatic Chromatin IP Kit (CST, Danvers, MA, USA), cells were crosslinked with 1% formaldehyde, and glycine was added to terminate the crosslinking. After cell lysis, chromatin DNA was broken down into fragments by ultrasound. Magnetic beads and anti‐MEF2A (1:50, A303‐531A, Thermo Fisher Scientific) antibody or anti‐IgG antibody (1:100, ab205718, Abcam) were added to the cell lysate and cultured at 4°C overnight. The protein‐DNA complexes were then eluted using magnetic beads, and the DNA products were analyzed using RT‐qPCR.

### 2.10 Dual‐Luciferase Reporter System

The JASPAR database revealed binding sites for MEF2A in the PSMD14 promoter region (https://jaspar.elixir.no/). The wild‐type (WT) or mutant (MUT) promoter regions of PSMD14 with binding sites to MEF2A were cloned onto the pGL3‐basic vectors to construct the luciferase reporter plasmid. After 12 h, cells were co‐transfected with the above plasmids and OE‐MEF2A or Vector‐NC for 48 h. Finally, luciferase activity was assessed using the dual luciferase report assay system.

### 2.11 Co‐Immunoprecipitation (Co‐IP) Assays

After cell lysis, the supernatant was obtained by centrifugation. The clarified cell lysate was then incubated with PSMD14 antibody (1:30, ab109130, Abcam) or the IgG antibody (1:100, ab205718, Abcam) as well as protein A/G beads at 4°C overnight. Subsequently, the antibody and protein complexes were separated by a magnetic rack, and then the proteins were denatured with 2 × SDS‐PAGE loading buffer (Beyotime, China) and identified using Western blot.

### 2.12 Ubiquitination Degradation Assay

To assess the effect of PSMD14 on RBM15B ubiquitination, 2 μg of sh‐PSMD14 and HA‐Ub plasmids were transfected into PANC‐1 and SW1990 cells, respectively. Then, the cells were treated with 5 mmol/L MG132, and levels of PSMD14 and RBM15B were then examined via Western blot using PSMD14 antibody (1:30, ab109130, Abcam) and RBM15B antibody (1:50, #25261, CST).

### 2.13 RNA Immunoprecipitation (RIP) Assay

RIP assay was performed following the protocol provided by the RNA Immunoprecipitation Kit (GENESEED, China). After transfection with Vector‐NC or OE‐RBM15B, cells were lysed. Then, the proteins were cultured overnight at 4°C with RBM15B antibody (1:50, #25261, CST) or IgG antibody (1:100, ab205718, Abcam). Protein A/G beads were added to the above samples and cultured for 1 h. Subsequently, RNA was extracted and purified. The SPON2 mRNA relative levels were analyzed by RT‐qPCR.

### 2.14 Methylated RNA Immunoprecipitation (MeRIP) Assay

Total RNA was extracted from PDAC and its adjacent non‐tumor tissues using Trizol reagent (Takara, China). MeRIP experiments were carried out following the instructions of the Magna MeRIP m6A Kit (Merck, Darmstadt, Germany). The total RNA was fragmented into oligonucleotides about 100‐nt long with fragmentation buffer at high temperature. Anti‐m6A antibody was pre‐bound to Protein A/G beads for 30 min. Samples were then inculcated with anti‐m6A antibody (1:200, ab151230, Abcam) or IgG antibody (1:200, ab205718, Abcam) at 4°C for 4 h. After washing, the buffer phenol‐chloroform was used for RNA extraction, and the enrichment of SPON2 mRNA was analyzed by RT‐qPCR.

### 2.15 SPON2 mRNA Stability Detection

Cells transfected with sh‐PSMD14 or OE‐RBM15B were supplemented with 5 μg/mL actinomycin D and cultured for 0, 2, 4, 6, and 8 h. Then total RNA was gathered. The levels of remaining SPON2 mRNA were detected by RT‐qPCR.

### 2.16 RT‐qPCR

Total RNA was obtained from samples with Trizol (Takara). PrimeScript RT Reagent Kit (Takara) was employed to synthesize cDNA. Then, MEF2A, PSMD14, and SPON2 mRNA levels were measured with TB Green Premix Ex TaqII reagent (Takara). Gene expression was calculated by 2^−ΔΔCt^ with GAPDH as the internal reference. Primer sequences were shown in Table 2.

**TABLE 2:** The primer sequences in RT‐qPCR (5′‐3′).

Gene	Forward	Reverse
MEF2A	AGCTGCTCCGGAGATACGAT	TCCGCCCCATTTTCAGTCAA
PSMD14	TCACCCTGGCTTTGGTTGTT	CACAGCTCTCTCCGACAAGG
SPON2	GCACGCTGGCTCTAATTTCG	AGAGCCCGGAGCTCCTATTT
GAPDH	CTGACTTCAACAGCGACACC	GTGGTCCAGGGGTCTTACTC

### 2.17 Western Blot

After cell lysis, the total protein was quantified with the BCA protein assay kit (Beyotime). The protein was subjected to SDS‐PAGE for separation and then transferred onto PVDF membranes at 4°C for 90 min. After treatment with skim milk, PVDF membranes were incubated with anti‐PSMD14 (1:2000, ab109130, Abcam), anti‐SPON2 (1:2000, ab302897, Abcam), anti‐RBM15B (1:1500, #25261, CST), and anti‐β‐actin (1 μg/mL, ab7817, Abcam) antibodies at 4°C overnight and then incubated with the secondary antibody (1:1000, ab205718, Abcam). The bands were visualized on the imaging system using enhanced chemiluminescence reagents and analyzed by Image J software.

### 2.18 Statistical Analyses

All experiments were conducted with a minimum of three independent biological replicates. Data were reported as mean ± standard deviation (SD). Statistical analysis was based on SPSS 20.0 software (Inc. Chicago, USA). Paired samples were analyzed using the paired *t*‐test. Differences between multiple groups were determined using the One‐way analysis of variance (ANOVA), followed by Tukey's post hoc test. The Kolmogorov–Smirnov test was performed for normality and equal variance of the data. Pearson's correlation analysis was used to determine the relationship between high and low PSMD14 expression and clinical characteristics of PDAC patients. A *p*‐value less than 0.05 (*p* < 0.05) was identified as statistically significant.

## 3 Results

### 3.1 PSMD14 Expression Was Upregulated in PC

**FIGURE 1:**
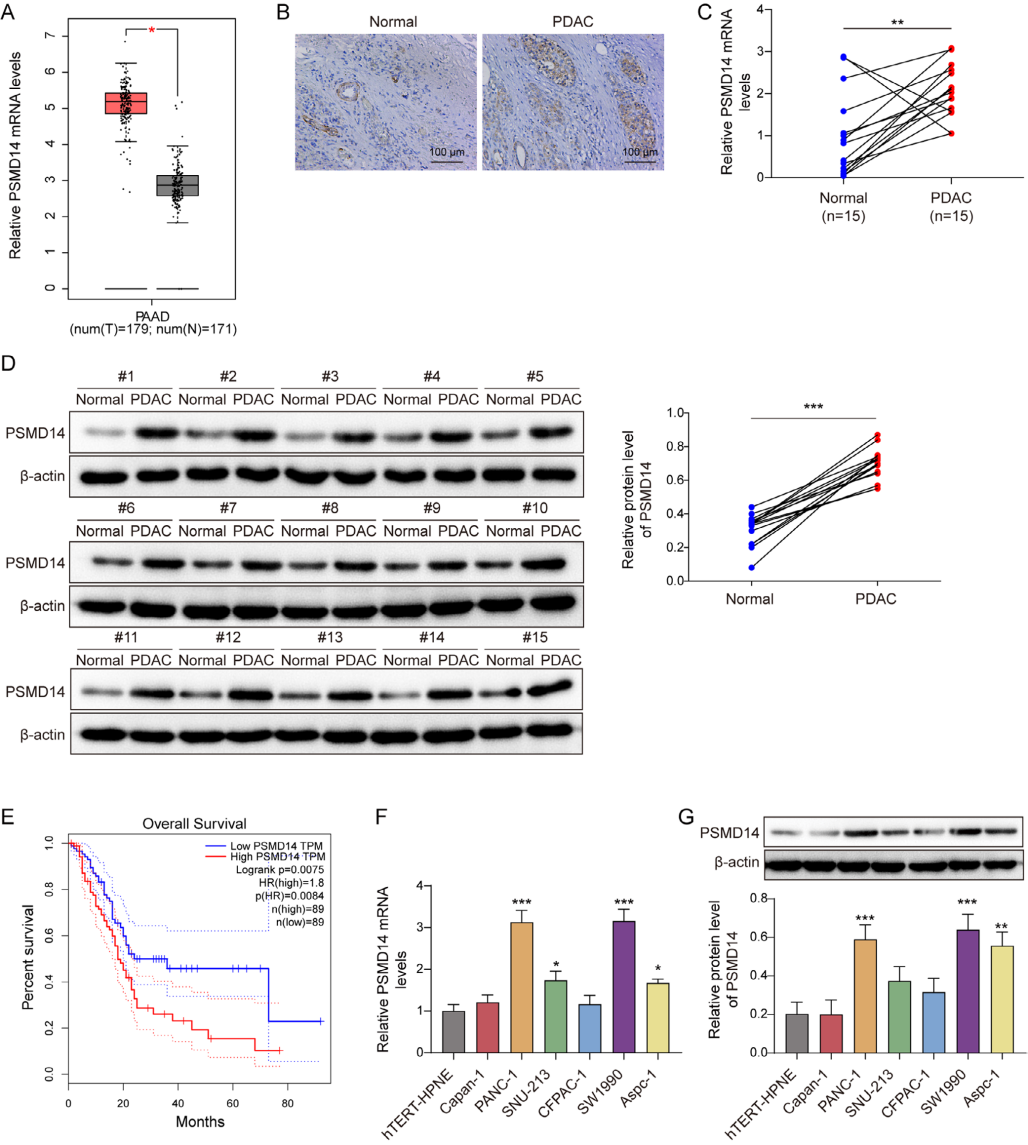
PSMD14 expression was upregulated in PC. Fifteen cases each of human PDAC tissues and adjacent non‐tumor tissues were collected. (A) PSMD14 expression in PDAC patients was predicted by GEPIA. (B) Detection of PSMD14 expression in clinical tissues using IHC (*n* = 15). (C, D) Levels of PSMD14 mRNA or protein in clinical tissues were checked by RT‐qPCR (*n* = 15) or Western blot (*n* = 5). (E) The association between high and low PSMD14 expression and poor prognosis in PAAD patients predicted using GEPIA. (F, G) RT‐qPCR or Western blot was performed to test for PSMD14 in hTERT‐HPNE, Capan‐1, PANC‐1, SNU‐213, CFPAC‐1, SW1990, and Aspc‐1 (*n* = 3). *p < 0.05, **p < 0.01, and ***p < 0.001.

In our study, GEPIA predicted that a marked upregulation of PSMD14 expression in human pancreatic adenocarcinoma (PAAD) tissues (Figure 1A). In PDAC clinical tissues, PSMD14 expression markedly elevated and was primarily localized in the cytoplasm (Figure 1B–D). Moreover, PAAD patients with high PSMD14 expression had poor prognosis (Figure 1E). According to the median PSMD14 expression was categorized into high and low expression, and the relationship between high and low PSMD14 expression and clinical characteristics of PDAC patients was analyzed in Table 1. The results revealed that PSMD14 had significant differences in Lymph node metastasis and TNM stage. Meanwhile, PSMD14 protein and mRNA levels were upregulated in PC cell lines compared to hTERT‐HPNE, with the most significant upregulation in PANC‐1 and SW1990 cells (Figure 1F,G). The above results suggested that PSMD14 was correlated with PC progression.

### 3.2 Knockdown of PSMD14 Inhibited Proliferation, Migration, and Invasion of PC

**FIGURE 2:**
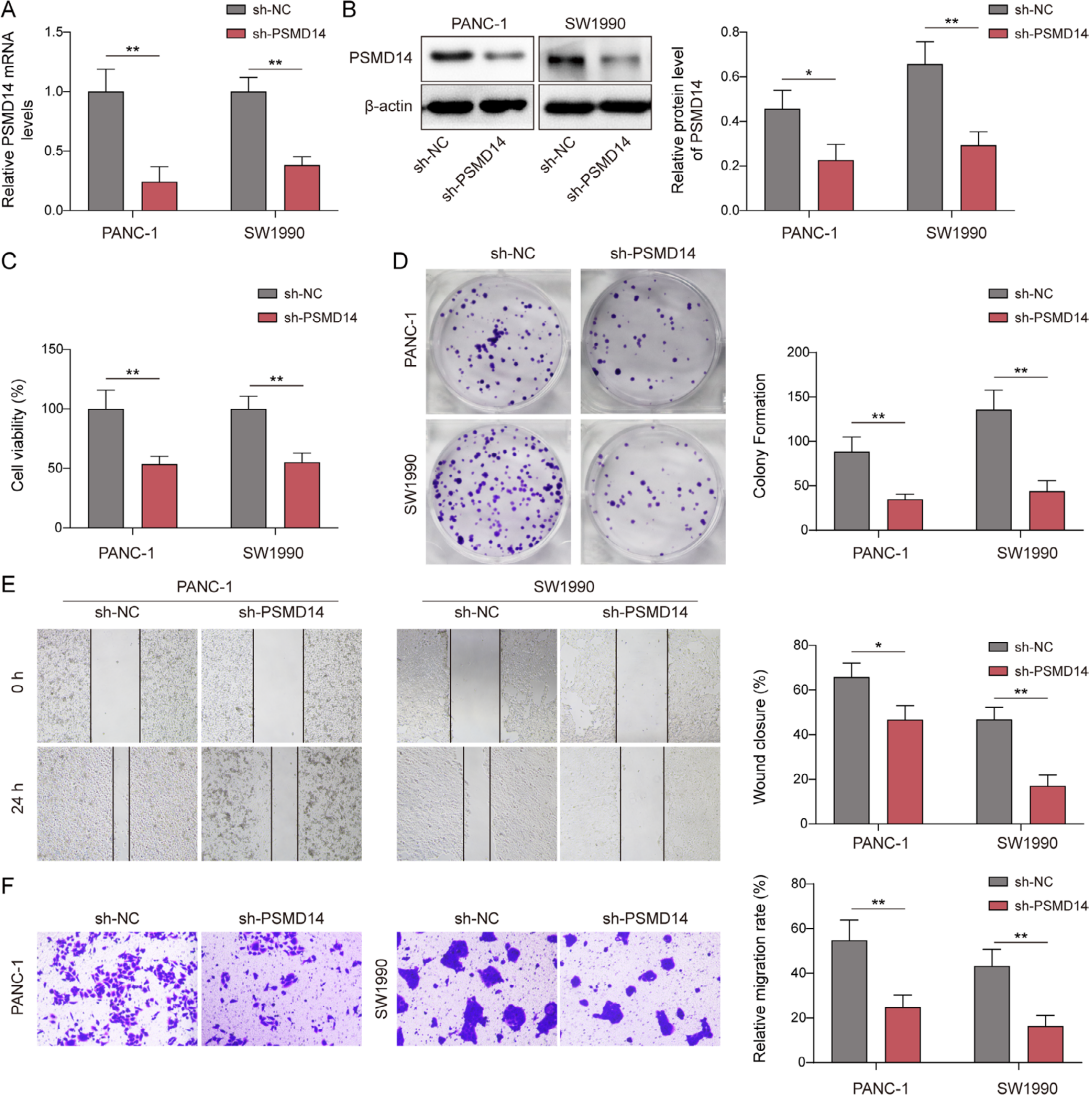
Knockdown of PSMD14 inhibited proliferation, migration, and invasion of PC. The sh‐PSMD14 vector was transfected into PANC‐1 and SW1990 cells. (A, B) RT‐qPCR or Western blot was checked PSMD14 expression in PC cells. (C) Viability of cells were tested by CCK‐8 kits. D. Clone formation assay was performed to assess cell proliferation. (E) Attenuation of cell migration was tested by scratch wound healing assay. (F) Transwell for detecting invasion ability. *n* = 3. *p < 0.05, **p < 0.01, and ***p < 0.001.

The role of PSMD14 in PC progression was further investigated. These results showed that transfection of sh‐PSMD14 resulted in a significant decrease in PSMD14 levels in PC cells (Figure 2A,B), indicating that PSMD14 was successfully knocked down. Knockdown of PSMD14 reduced PC cell viability (Figure 2C). At the same time, the proliferation, migration, and invasion abilities were diminished after the knockdown of PSMD14 (Figure 2D–F). Therefore, the knockdown of PSMD14 effectively inhibited PC development.

### 3.3 MEF2A Activated PSMD14 Transcription

**FIGURE 3:**
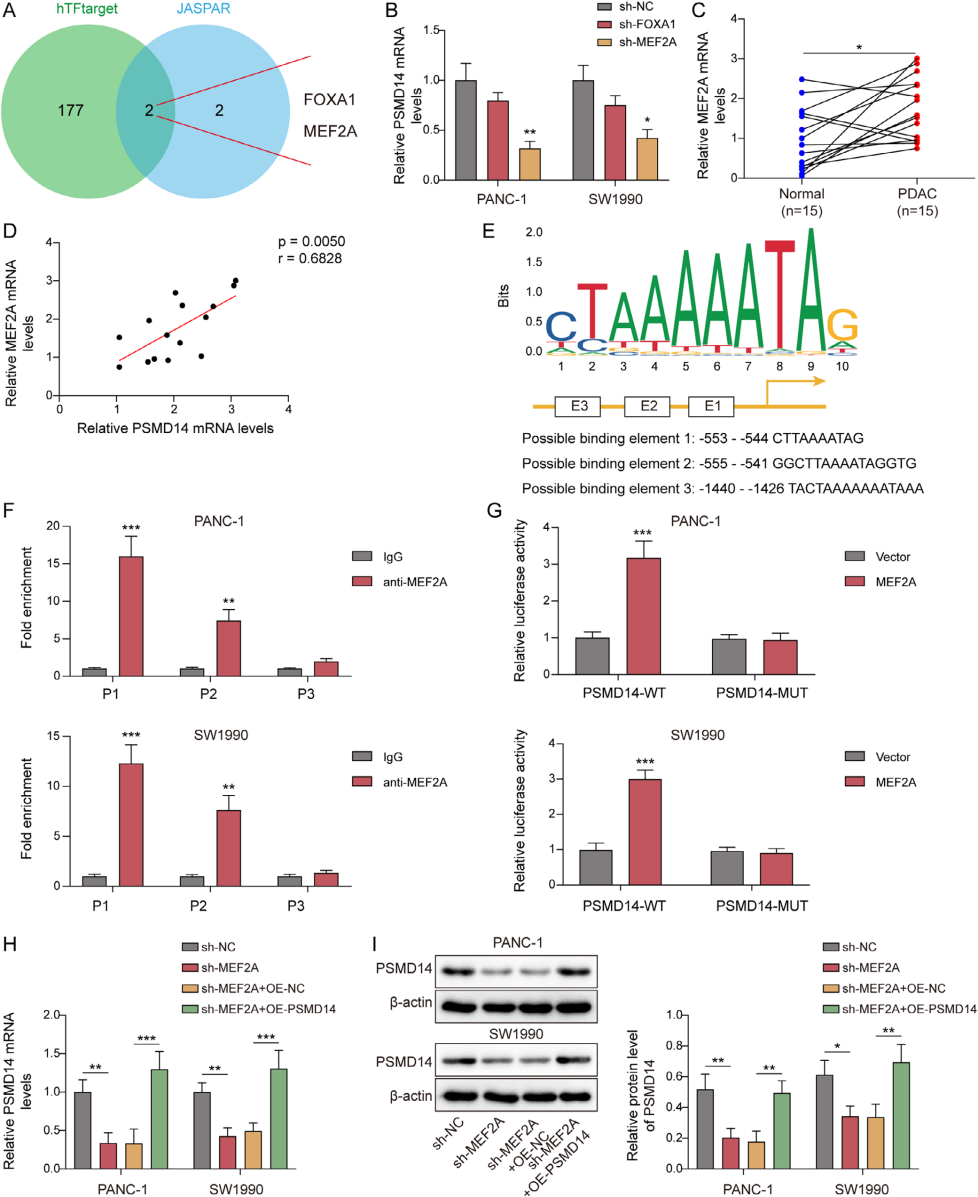
MEF2A activated PSMD14 transcription. (A) Upstream transcription factors of PSMD14 were screened by bioinformatics. (B) RT‐qPCR was used to check PSMD14 level after knockdown of MEF2A or FOXA1 in PANC‐1 and SW1990 cells (*n* = 3). (C) MEF2A levels was detected in human PDAC tissues and neighboring non‐tumor tissues by RT‐qPCR (*n* = 15). (D) MEF2A and PSMD14 correlation analysis. (E) JASPAR predicted the binding sites of MEF2A and PSMD14. (F, G) ChIP or dual luciferase assay verified the MEF2A and PSMD14 interactions (*n* = 3). (H, I) After transfection of sh‐MEF2A and OE‐PSMD14, PSMD14 expression was examined via RT‐qPCR or Western blot in PANC‐1 and SW1990 cells (*n* = 3). *p < 0.05, **p < 0.01, and ***p < 0.001.

Subsequently, this study investigated the upstream mechanism of PSMD14. First, there are two transcription factors (MEF2A and FOXA1) of PSMD14 were discovered through bioinformatics analysis (Figure 3A). After transfecting with sh‐FOXA1 or sh‐MEF2A, the expression of PSMD14 was downregulated, with the most significant downregulation in the sh‐MEF2A group in PC cells (Figure 3B). Moreover, MEF2A mRNA levels were increased in human PDAC tissues (Figure 3C). Notably, MEF2A and PSMD14 were positively correlated in PDAC (Figure 3D). Importantly, JASPAR predictions displayed that there were potential binding sites between the MEF2A and PSMD14 promoter regions (Figure 3E). ChIP assay revealed that PSMD14 was greatly enriched after immunoprecipitation with anti‐MEF2A antibodies at the P1 site, which indicated that P1 was confirmed as an interaction site between MEF2A and PSMD14 (Figure 3F). Also, overexpression of MEF2A remarkably increased luciferase activity of PSMD14‐WT (Figure 3G), further confirming the regulatory role of MEF2A and PSMD14 at the P1 site. Finally, PSMD14 expression was downregulated after the knockdown of MEF2A, while overexpression of PSMD14 attenuated this change (Figure 3H,I). These results revealed that MEF2A transcription upregulated PSMD14 expression.

### 3.4 PSMD14 Upregulated SPON2 Expression in an m6A‐RBM15B‐Dependent Manner

**FIGURE 4:**
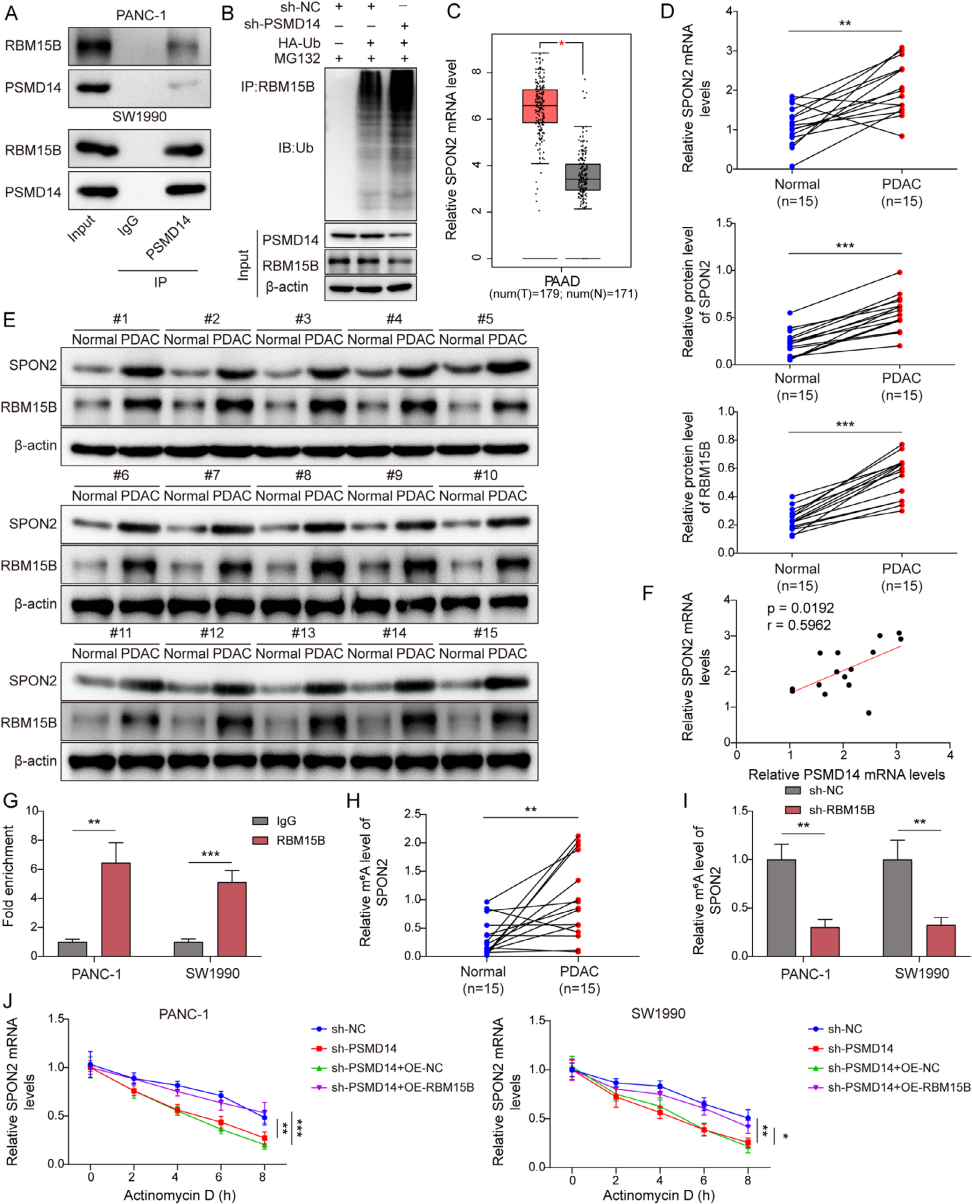
PSMD14 upregulated SPON2 expression in an m6A‐RBM15B‐dependent manner. (A) In PANC‐1 and SW1990 cells, the interaction between PSMD14 and RBM15B was verified by Co‐IP (*n* = 3). (B) After transfection of sh‐PSMD14 and HA‐Ub, PSMD14, and RBM15B levels in PC cells were examined by Western blot (*n* = 3). (C) GEPIA predicted SPON2 expression in PC. (D) Detection of SPON2 expression in clinical tissues by RT‐qPCR (*n* = 15). (E) Analysis of SPON2 and RBM15B protein levels in human PDAC and adjacent non‐tumor tissue by Western blot (*n* = 5). (F) PSMD14 and SPON2 correlation analysis (*n* = 15). (G) RIP assay for validation of the interaction between RBM15B and SPON2 (*n* = 3). H. MeRIP assay was detected SPON2 m6A levels in human PDAC tissues and adjacent non‐tumor tissues (*n* = 15). (I) After knockdown RBM15B, SPON2 m6A levels were examined using MeRIP in PANC‐1 and SW1990 cells (*n* = 3). (J) Sh‐PSMD14 and OE‐RBM15B were transfected into PANC‐1 and SW1990 cells, respectively, and then treated with actinomycin D, and SPON2 mRNA stability was tested by RT‐qPCR (*n* = 3). *p < 0.05, **p < 0.01, and ***p < 0.001.

Next, we explored the downstream mechanisms of PSMD14. IntAct predictions indicated that there were potential binding sites between PSMD14 and RBM15B, and Co‐IP assay further validated their interaction (Figure 4A). Importantly, the knockdown of PSMD14 resulted in elevated levels of RBM15B ubiquitination and downregulated expression of RBM15B (Figure 4B). Then, the GEPIA predictions revealed that SPON2 expression was upregulated in PC (Figure 4C). In human PDAC tissue, the expression of SPON2 and RBM15B was significantly increased (Figure 4D,E), and PSMD14 was found to positively correlate with SPON2 (Figure 4F). More importantly, Starbase predicted that RBM15B was an RNA‐binding protein of SPON2. In the RIP assay, SPON2 mRNA was markedly enriched after immunoprecipitation with anti‐RBM15B antibody, demonstrating a binding interaction between RBM15B and SPON2 (Figure 4G). It was known that RBM15B mediated m6A gene modification [18]. Notably, the SRAMP prediction discovered the presence of multiple m6A modification sites in SPON2. The MeRIP assay further validated that the m6A levels of SPON2 were upregulated in human PDAC tissues (Figure 4H). Furthermore, SPON2 mRNA levels were decreased in PC cells following RBM15B knockdown (Figure 4I). Finally, PSMD14 knockdown promoted SPON2 mRNA degradation in actinomycin D‐treated PC cells, while the overexpression RBM15B inhibited SPON2 mRNA degradation (Figure 4J). Therefore, PSMD14 upregulated SPON2 expression in an m6A‐RBM15B‐dependent manner.

### 3.5 PSMD14 Promoted PC Proliferation, Migration, and Invasion by Upregulating SPON2

**FIGURE 5:**
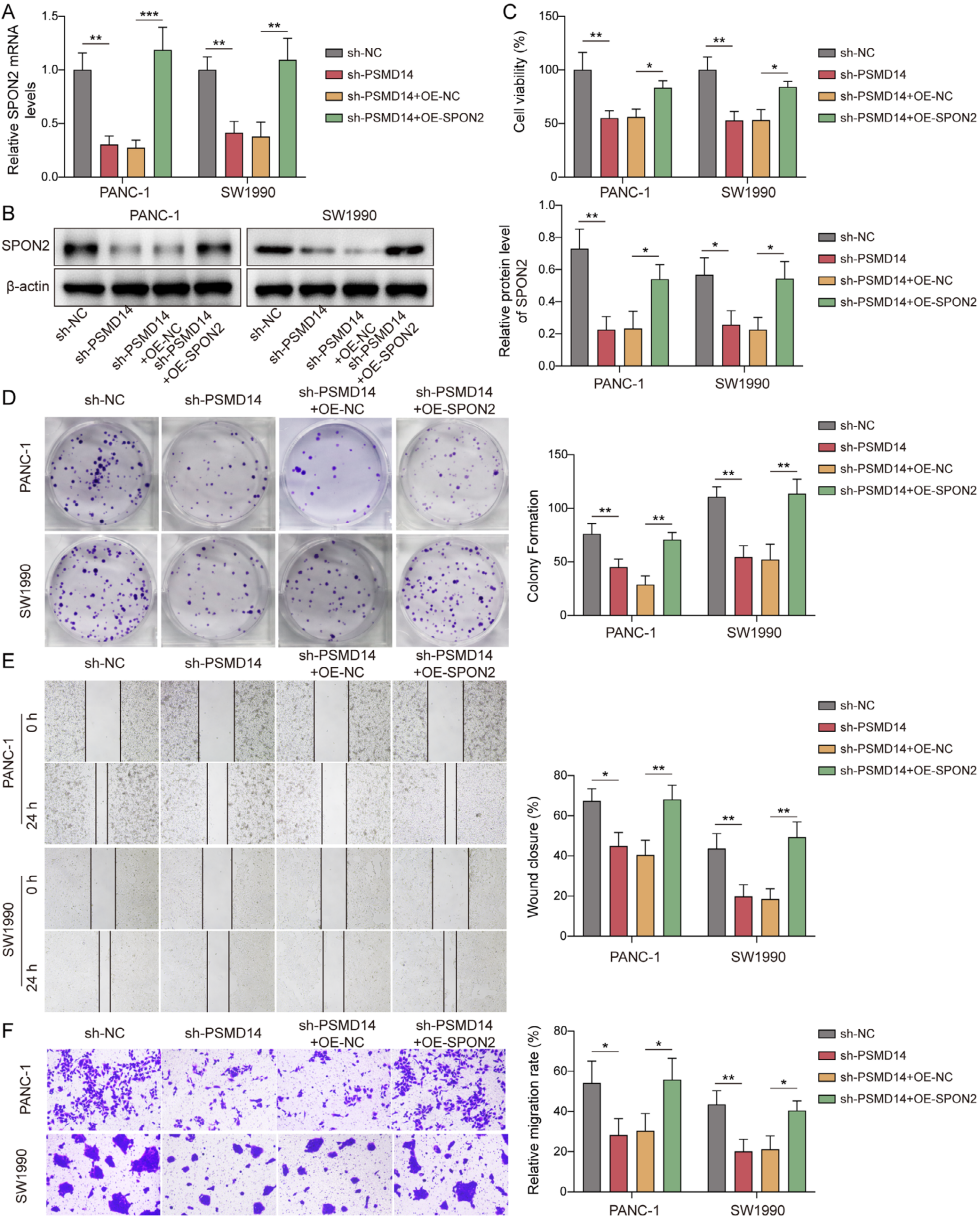
PSMD14 promoted PC proliferation, migration, and invasion by upregulating SPON2. The sh‐PSMD14 and OE‐SPON2 were transfected into PANC‐1 and SW1990 cells. (A) RT‐qPCR was employed to determine SPON2 mRNA expression. (B) Western blot was carried out to analyze SPON2 expression. (C) Cell viability was detected by CCK‐8. (D) Clone formation assay was implemented to test for cell proliferation. (E) Cell migration was measured by scratch wound healing assay. (F) Transwell for testing cell invasion ability. *n* = 3. *p < 0.05, **p < 0.01, and ***p < 0.001.

Subsequently, this study further investigated whether PSMD14 regulated PC proliferation, migration, and invasion through SPON2. It was observed that PSMD14 knockdown decreased SPON2 mRNA and protein levels in PC cells, while SPON2 overexpression reversed this change (Figure 5A,B). Besides, overexpression of SPON2 alleviated the decrease in PC cell viability caused by the knockdown of PSMD14 (Figure 5C). Meanwhile, overexpression of SPON2 promoted proliferation, migration, and invasion of PC cells, which weakened the effect of the knockdown of PSMD14 (Figure 5D–F). As a result, PSMD14 promoted proliferation, migration, and invasion of PC cells by upregulating SPON2.

### 3.6 Knockdown of PSMD14 Inhibited Tumor Growth and Lung Metastasis of PC In Vivo

**FIGURE 6:**
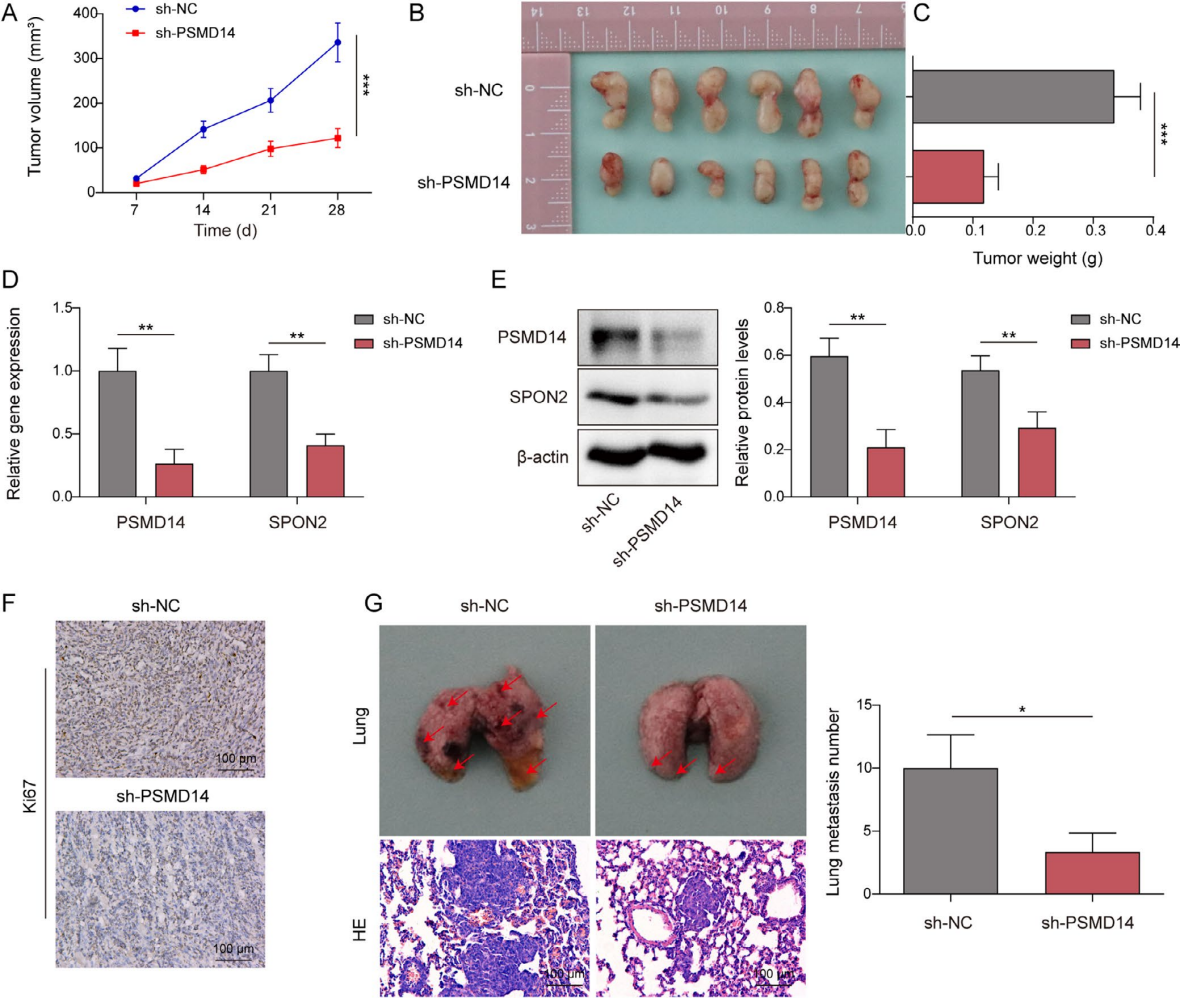
Knockdown of PSMD14 inhibited tumor growth and lung metastasis of PC in vivo. PANC‐1 cells transfected with sh‐PSMD14 were injected subcutaneously into BALB/c‐nu/nu mice to construct a PC subcutaneous transplanted tumor model. (A–C) Subcutaneous tumor volumes, gross body maps, and weights of mice were recorded and analyzed. (D, E) Expression of PSMD14 and SPON2 in subcutaneous tumor tissues was investigated by RT‐qPCR or Western blot. (F) Expression of KI67 was measured by IHC to assess tumor proliferation. (G) After intravenous injection of PANC‐1 cells transfected with sh‐PSMD14 in BALB/c‐nu/nu mice, lung tissue was photographed and recorded. Then, lung tissue was stained with HE for metastatic nodule analysis. *n* = 6. *p < 0.05, **p < 0.01, and ***p < 0.001.

The role of PSMD14 in PC mice was evaluated. PANC‐1 cells transfected with sh‐PSMD14 were injected subcutaneously into mice. First, PSMD14 knockdown significantly reduced PC tumor volume and weight (Figure 6A–C). When mice were injected with sh‐PSMD14, the expression of PSMD14 and SPON2 was reduced (Figure 6D,E). Additionally, IHC results further exhibited that PSMD14 knockdown decreased expression of the proliferation marker Ki‐67 (Figure 6F). To assess lung metastasis, PANC‐1 cells transfected with sh‐PSMD14 were injected into the tail vein of mice. It was observed that PSMD14 knockdown inhibited lung metastasis and reduced the number of metastatic nodules in mice's lungs (Figure 6G). These results demonstrated that PSMD14 knockdown prevented tumor growth and lung metastasis in vivo.

## 4 Discussion

PC is the “king of cancer,” with extremely high malignancy and poor prognosis [3]. The main reasons for the poor prognosis of PC include easy metastasis, high recurrence rate, and drug resistance [28]. Therefore, there is a need to find new biomarkers and further investigate the molecular mechanisms of PC progression to address this issue. In our study, we emphasized the importance of PSMD14 for PC progression and revealed that MEF2A transcriptional activation of PSMD14 upregulated SPON2 expression in an m6A‐RBM15B‐dependent manner, which promoted PC cell proliferation, invasion, and migration.

PSMD14 is involved in critical physiological and pathological events, including tumor growth [29]. Previous studies showed that PSMD14 promoted the progression of several cancers. For example, PSMD14 promoted positive breast tumor growth by stabilizing estrogen signaling [9]. PSMD14 expression was obviously elevated in osteosarcoma tissue, and PSMD14 knockdown effectively inhibited the proliferation, migration, and invasion of osteosarcoma cells [30]. Furthermore, Zhou et al. [12] predicted that PSMD14 was closely related to overall tumor survival in PDAC. One study claimed that PSMD14 promoted the growth of PDAC tumors by deubiquitinating MYC protein and potentiating acinar‐to‐ductal metaplasia and was a potential therapeutic target [10]. Also, clinical evidence from this study suggested that abnormal PSMD14 expression was positively associated with poor prognosis in PDAC patients [10]. Our clinical results were consistent with this. It indicated that high PSMD14 expression was associated with poor prognosis in PAAD patients and that PSMD14 expression showed significant differences in Lymph node metastasis and TNM stage. We discovered that PSMD14 expression was upregulated in human PDAC tissues and PC cells. More critically, knockdown of PSMD14 inhibited PC cell proliferation, migration, and invasion in vitro and suppressed PC tumor growth and lung metastasis in vivo. Thus, our current research proved for the first time that knockdown of PSMD14 inhibited PC development. In the future, we will collect more clinical samples to further analyze the relationship between PSMD14 and PDAC under clinical conditions, such as survival and prognosis analysis, ROC curve analysis, etc., aiming at laying the foundation for the clinical application of PSMD14, and then clarifying its clinical significance, such as therapeutic targets, prognostic markers, and so on.

Next, we investigated the upstream molecular targets of PSMD14. The role of transcription factors regulating gene transcription in cancer progression has attracted increasing attention [31]. A study revealed that estrogen receptor alpha bound to the PSMD14 promoter region and promoted the transcription of this gene, which promoted breast cancer progression [9]. Another study reported that RELA promoted the progression of myeloma through transcriptional activation of PSMD14 [8]. The above studies demonstrated that transcriptional activation of PSMD14 was closely associated with tumor progression. As we all know, MEF2A is a transcription factor that may influence tumor progression by regulating gene expression [12, 32]. For example, MEF2A promoted tumor proliferation and metastasis by transcriptionally upregulating the expression of ZEB2 and CTNNB1 in colorectal cancer [12]. In addition, only one study demonstrated that MEF2A was required for PC cell proliferation, but the expression level of MEF2A in PCs was not clear [15]. In this study, we first identified significant high expression of MEF2A in PC and elucidated the transcriptional activation of PSMD14 by MEF2A.

Subsequently, we explored the downstream molecular mechanisms by which PSMD14 promoted PC progression. PSMD14 is a member of the JAB1/MPN+/MOV34 domain protease family of deubiquitinating enzymes [33]. Numerous studies have revealed the role of PSMD14 in inhibiting protein degradation through ubiquitination in cancer. Studies showed that PSMD14 promoted hepatocellular carcinoma growth and metastasis by inhibiting the ubiquitination of growth factor receptor‐bound protein 2 [23]. Yang et al. [9] found a new positive feedback loop between PSMD14 and ERα signaling, where PSMD14 inhibited ERα ubiquitination, and ERα could bind to the promoter region of PSMD14 and promote its gene transcription, which in turn contributed to breast cancer development. It was also discovered that PSMD14 decreased PKM2 ubiquitination, downregulated the ratio of PKM2 tetramers to dimers and monomers, which in turn decreased pyruvate kinase activity, induced nuclear translocation of PKM2, and promoted ovarian development [33]. Consequently, PSMD14 could promote cancer development through multiple pathways. Here, we also confirmed that PSMD14 mediated deubiquitination of RBM15B to increase RBM15B protein expression. RBM15B was increased in human PDAC tissues. RBM15B is one of the most critical m6A methyltransferases that selectively splices mRNAs and acts as an mRNA export factor in tumorigenesis and development [20]. According to the report, RBM15B enhanced TRAM2 mRNA stability by relying on its m6A methyltransferase activity in the TRAM2 3'‐UTR, thereby promoting hepatocellular carcinoma proliferation [19]. Another study indicated that glutathione peroxidase 4 (GPX4) activated stimulator of interferon (STING) genes through RBM15B‐mediated m6A modification, thereby promoting an anti‐tumor immune response in colon adenocarcinoma hosts [34]. Studies have shown that RBM15B has been identified as a high‐confidence interactor with Wilms' tumor 1‐associating protein (WTAP) [35]. While WTAP is able to bind to methyltransferase like 3 (METTL3) [36]. METTL3 is a methyltransferase that mediates the methylation of m6A in mRNA, and is recruited to RNA by an unknown adaptor protein to m6A modification [37]. A study showed that RBM15B were able to recruit the m6A methylation complex Wilms' tumor 1‐associating protein (WTAP)‐METTL3 (methyltransferase like 3) to specific sites in long non‐coding RNA X‐inactive specific transcript (XIST), thereby promoting its m6A modification [37]. In our research, we first showed that PSMD14 upregulated SPON2 mRNA expression in an m6A‐RBM15B‐dependent manner. Whether RBM15B affects SPON2 mRNA m6A modification by recruiting the WTAP‐METTL3 complex and thus unknown. The specific molecular mechanism by which RBM15B regulates SPON2 needs to be further verified in the future. A series of reports have revealed that SPON2 promoted many tumors progression, including hepatocellular carcinoma, gastric cancer, and lung adenocarcinoma [38, 39, 40]. We identified for the first time that PSMD14 promoted PC proliferation, invasion, and migration by upregulating SPON2 expression. In short, our results confirmed that MEF2A transcriptionally activated PSMD14 and that PSMD14 drove the PC disease process by elevating SPON2 expression in an m6A‐RBM15B‐dependent manner.

In sum, our study suggested that PSMD14 knockdown inhibited PC progression. We further revealed the molecular mechanism that MEF2A transcriptionally activated PSMD14, and upregulated PSMD14 promoted SPON2 mRNA stability in an m6A‐RBM15B‐dependent manner by repressing ubiquitination and degradation of RBM15B. These findings may provide new mechanisms and therapeutic targets for early diagnosis of PC.

## Conflicts of Interest

The authors declare no conflicts of interest.

## Data Availability

The data that support the findings of this study are available from the corresponding author upon reasonable request.
